# Effects of Acid Etching and Bleaching on Enamel Stain Retention From Turmeric and Coffee: An In Vitro Study

**DOI:** 10.7759/cureus.90134

**Published:** 2025-08-15

**Authors:** Sachin Kandalkar, Shreya Bhukal, Apurva R Kamble, Nikhil Sathawane, Gaurav Nagar, Manish Sharma

**Affiliations:** 1 Department of Oral Pathology, Sahkar Maharshi Bhausaheb Thorat Dental College and Hospital, Sangamner, IND; 2 Department of Public Health Dentistry, All India Institute of Medical Sciences, New Delhi, New Delhi, IND; 3 Department of Conservative Dentistry and Endodontics, D Y Patil Dental School, Pune, IND; 4 Department of Conservative Dentistry and Endodontics, Swargiya Dadasaheb Kalmegh Smruti Dental College and Hospital, Nagpur, IND; 5 Department of Periodontics, Manav Rachna Dental College, Faridabad, IND; 6 Department of Oral Pathology, Jawahar Medical Foundation - Annasaheb Chudman Patil Memorial Dental College, Dhule, IND

**Keywords:** bleaching, discoloration, enamel, etching, staining

## Abstract

Introduction: Dental procedures, such as acid etching and bleaching, are widely used to improve the appearance of teeth. However, dietary chromogens, such as turmeric and coffee, may affect the aesthetic outcomes of these treatments by staining the enamel. Understanding how these procedures influence the interaction between enamel and common staining agents is essential for optimizing dental aesthetics. This in vitro study compared the effects of 37% phosphoric acid etching, 10% hydrogen peroxide bleaching, and their combination on enamel surface roughness and stain retention when exposed to turmeric and coffee, simulating clinical conditions and dietary exposures.

Materials and methods: Sixty non-carious human maxillary premolars were sectioned at the cementoenamel junction, and the buccal surfaces were ground flat to create a standardized 5 mm × 5 mm enamel surface. The specimens were randomly divided into three groups (n = 20 each): Group A (control, no etching), Group B (etched for 30 seconds with 37% phosphoric acid gel; Scotchbond Etchant, 3M Company, St. Paul, MN), and Group C (treated with 10% hydrogen peroxide gel for 10 minutes; Opalescence Boost, Ultradent Products, Inc., South Jordan, UT). Each group was subdivided into two subgroups (n = 10) for staining with either turmeric (10 g McCormick Ground Turmeric, McCormick & Company, Hunt Valley, MD) in 100 mL distilled water or coffee (10 g Nescafé Classic, Nestlé S.A., Vevey, Switzerland) in 100 mL distilled water at 37°C for 10 minutes, three times daily for seven days. Surface roughness (Ra and Rz) was measured using a profilometer (Surftest SJ-210, Mitutoyo Corporation, Kawasaki, Japan) before and after staining. Stain retention was quantified via a chemical staining assay using a UV-vis microplate reader (Synergy H1; BioTek Instruments, Inc., Winooski, VT) at 425 nm for turmeric and 275 nm for coffee.

Results: Phosphoric acid etching resulted in significantly higher stain retention (coffee: 25.60 ± 2.10 µg/mL; turmeric: 16.80 ± 1.8 µg/mL) than hydrogen peroxide (coffee: 18.20 ± 1.50 µg/mL; turmeric: 12.70 ± 1.20 µg/mL) and control (coffee: 8.30 ± 0.90 µg/mL; turmeric: 5.10 ± 0.60 µg/mL) (p < 0.001). Surface roughness increased significantly after staining in all groups (p < 0.05), with coffee causing greater changes than turmeric. The phosphoric acid-etched surfaces exhibited the highest roughness (coffee: 2.38 µm; turmeric: 2.05 µm).

Conclusion: Phosphoric acid etching significantly enhanced enamel staining susceptibility and roughness compared to hydrogen peroxide bleaching, with coffee posing a greater risk than turmeric. These findings underscore the need for dietary counseling and post-treatment protective measures to preserve dental aesthetics.

## Introduction

Dental aesthetics play a critical role in patient satisfaction and overall oral health perception, with tooth discoloration being a common concern that prompts individuals to seek dental intervention [1]. Enamel, the hardest tissue in the human body, is susceptible to extrinsic staining from dietary substances such as turmeric and coffee, which are widely consumed [2]. Turmeric, derived from the rhizome of *Curcuma longa*, contains curcumin, a polyphenolic compound known for its vibrant yellow pigment and potential to adhere to dental surfaces [3]. Coffee, which is rich in chromogens such as caffeic acid derivatives, is another potent staining agent owing to its frequent consumption and chemical interaction with enamel [4]. These stains can compromise the aesthetic appeal of teeth, particularly when enamel surfaces are altered by dental procedures such as etching or bleaching, which are common in restorative and cosmetic dentistry [3].

Enamel etching with 37% phosphoric acid is a standard procedure in adhesive dentistry and is used to create micropores that enhance the bonding of restorative materials [5]. However, this process increases surface roughness, potentially enhancing stain retention by providing more sites for chromogen adhesion [6]. Similarly, hydrogen peroxide at a concentration of 10% is widely used for at-home bleaching of whitened teeth by the oxidation of organic pigments [7]. However, bleaching may alter the surface properties of enamel, including microhardness and susceptibility to staining, owing to the oxidative effects on the enamel matrix [7]. The combined use of phosphoric acid etching and hydrogen peroxide bleaching, often observed in clinical practice, may further modify the enamel surface characteristics, potentially exacerbating stain uptake. Despite the widespread use of these agents, their comparative impact on enamel susceptibility to staining by dietary substances such as turmeric and coffee remains unexplored.

Previous studies have investigated the staining potential of coffee and turmeric on natural and restored enamel [3,4], but few have focused on the effects on etched or bleached enamel [8]. For instance, Adeyemi et al. [9] reported that bleaching of enamel did not cause susceptibility of enamel to extrinsic staining. However, these studies often rely on visual or photographic assessments, which are subjective and prone to variability [3,4,8]. Quantitative methods, such as chemical staining assays, offer a more objective approach for measuring stain concentration without requiring images, thus improving reproducibility [10]. Understanding how etching and bleaching affect the interaction between enamel and dietary stains is crucial for developing strategies to minimize discoloration and optimize aesthetic outcomes in dental practice.

This in vitro study aimed to compare the effects of 37% phosphoric acid etching, 10% hydrogen peroxide bleaching, and their combination on the susceptibility of enamel surfaces to turmeric and coffee stains. Surface roughness, microhardness, and stain retention were evaluated using a profilometer, Vickers microhardness tester, and chemical staining assay, respectively, to provide quantitative insights. By simulating clinical conditions and dietary exposure, this study aimed to elucidate the interplay between enamel surface modifications and staining, contributing to evidence-based approaches for maintaining dental aesthetics.

## Materials and methods

Study design and setting

The study was designed as an in vitro study conducted at the Department of Conservative Dentistry and Endodontics, Jawahar Medical Foundation's Annasaheb Chudaman Patil Memorial Dental College, Dhule, India. The investigation spanned from March 2023 to May 2023, encompassing the sample preparation, etching, staining, and evaluation phases. Ethical approval was obtained from the Institutional Ethics Committee (EC/INST/2022/2959/SS234) prior to the collection of extracted human premolars, ensuring compliance with the ethical guidelines for the use of human biological materials.

Sample preparation

A power analysis conducted using G*Power (version 3.1.9.7; Heinrich-Heine-Universität Düsseldorf, Düsseldorf, Germany) with an F-test family (ANOVA, omnibus) approach, employing an effect size of 0.40, an alpha level of 0.05, and a power of 0.80 across three groups, indicated that a total of 60 samples (20 per group) provided 81.3% power to identify significant differences. The effect size was estimated based on pilot data on surface roughness (ΔRa ranging from 0.38 to 0.48 µm, with a standard deviation of 0.15-0.32).

**Figure 1:**
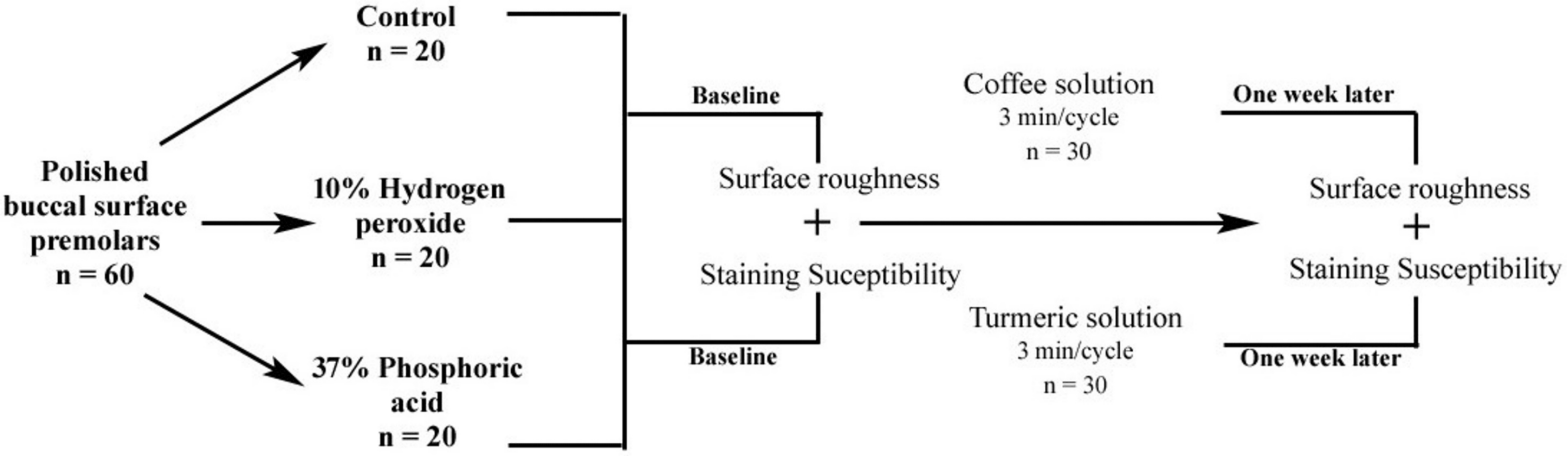
Study flow diagram.

Sixty freshly extracted, non-carious human maxillary premolars obtained for orthodontic reasons were collected and stored in 0.1% thymol solution at 4°C until use. The teeth were cleaned of debris and calculus using a scaler and polished with non-fluoridated pumice paste (pumice powder; Kerr Corporation, Orange, CA) using a rubber cup attached to a slow-speed handpiece (NSK EX-203; Nakanishi Inc., Kanuma, Japan). Each tooth was sectioned at the cementoenamel junction using a diamond disc (DiaTessin; Kerr Corporation, Orange, CA) to separate the crown. The buccal surface of each crown was ground flat using 600-grit silicon carbide paper (3M Wetordry; 3M Company, St. Paul, MN) to create a standardized enamel surface of approximately 5 mm × 5 mm. The prepared specimens were randomly divided into four groups (n = 20 per group): Group A (control, no etching), Group B (etched with 37% phosphoric acid) [5], and Group C (etched with 10% hydrogen peroxide) [11]. For Group B, the enamel surfaces were etched with 37% phosphoric acid gel (Scotchbond Etchant; 3M Company, St. Paul, MN) for 30 seconds, followed by rinsing with distilled water for 20 seconds and air-drying with an oil-free air syringe. For Group C, the enamel surfaces were treated with 10% hydrogen peroxide gel (Opalescence Boost; Ultradent Products, Inc., South Jordan, UT) for 10 minutes, followed by rinsing with distilled water for 20 seconds and air-drying. Group A received no treatment and was stored in artificial saliva (Biotene Oral Balance; GlaxoSmithKline, Brentford, UK) during the procedures (Figure 1).

Staining procedure

Each group was further subdivided into two subgroups (n = 10 each) for staining with turmeric or coffee. The turmeric solution was prepared by dissolving 10 g of turmeric powder (ground turmeric; McCormick & Company, Hunt Valley, MD) in 100 mL of distilled water and was heated to 37°C to simulate oral conditions. The coffee solution was prepared by dissolving 10 g of instant coffee (Nescafé Classic, Nestlé S.A., Vevey, Switzerland) in 100 mL of distilled water at 37°C. Each specimen was immersed in 50 mL of the respective staining solution (turmeric or coffee) for three minutes three times daily for seven days. The specimens were stored in artificial saliva (Biotene Oral Balance; GlaxoSmithKline, Brentford, UK) at 37°C in an incubator (Heratherm; Thermo Fisher Scientific, Waltham, MA) to mimic oral conditions. After each immersion, the specimens were rinsed with distilled water for 10 seconds to remove the excess stain.

Surface roughness evaluation

The enamel surface roughness was measured using a stylus profilometer (Surftest SJ-210; Mitutoyo Corporation, Kawasaki, Japan) to assess the changes caused by staining. Measurements were taken at baseline (pre-staining) and after the seven-day staining period (post-staining). The profilometer was calibrated to measure the roughness parameters Ra (average roughness) and Rz (maximum height of the profile) over a 4-mm evaluation length, with a cutoff of 0.8 mm. Three measurements were taken at different locations on each specimen, and the mean values were calculated. The profilometer stylus moved at a speed of 0.5 mm/s with a force of 0.75 millinewtons (mN) to ensure consistent contact with the enamel surface.

Color stability assessment

**Figure 2:**
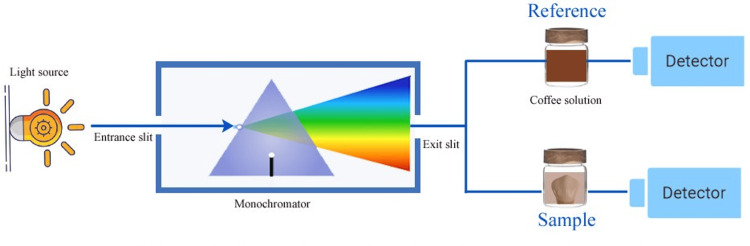
Detection of absorbance of the samples by UV-vis spectrophotometry. Reference absorbance for coffee was 425 nm, and that for turmeric was 275 nm. Original image created by the author.

Color changes on the enamel surfaces were evaluated using a chemical staining assay to quantify the retention of turmeric and coffee stains in µg/mL (micrograms per mL). After the seven-day staining period, each specimen was immersed in 10 mL of a 0.1 M sodium hydroxide solution (Sodium Hydroxide Pellets; Sigma-Aldrich, St. Louis, MO) for five minutes at 37°C to extract surface-bound stains. The solution was agitated gently using a vortex mixer (Vortex-Genie 2; Scientific Industries, Inc., Bohemia, NY) to ensure uniform extraction. The supernatant was collected, and the absorbance of the extracted stain was measured using a UV-vis microplate reader (Synergy H1; BioTek Instruments, Inc., Winooski, VT) at 425 nm for turmeric (corresponding to the peak absorbance of curcumin) and 275 nm for coffee (corresponding to caffeic acid derivatives) (Figure 2).

Calibration curves were prepared using known concentrations of turmeric powder and instant coffee dissolved in 0.1 M sodium hydroxide to convert absorbance readings to stain concentration (µg/mL) by equation: y = 0.034x + 0.02 (R² = 0.998), where y = absorbance (425 nm) and x = concentration (µg/mL) [10]. Measurements were taken in triplicate for each specimen, and the mean stain concentration was calculated. Baseline measurements were performed on unstained specimens after treatment (or storage in artificial saliva for Group A) to account for any intrinsic enamel absorbance. The difference in stain concentration between the baseline and post-staining measurements was used to quantify stain retention.

To ensure the reliability of the chemical staining assay, intra- and inter-examiner reliabilities were assessed for absorbance measurements. Ten randomly selected specimens from each staining subgroup (turmeric and coffee) were measured twice by the same examiner (intra-examiner reliability) with a 24-hour interval between the measurements. Additionally, a second examiner independently measured the same specimens to assess inter-examiner reliability. The absorbance readings were converted to stain concentrations (µg/mL) using calibration curves. The intraclass correlation coefficient (ICC) was calculated to evaluate the consistency of the measurements, with ICC values above 0.8 indicating excellent reliability. This assessment confirmed the reproducibility of the staining assay results across repeated measurements by different examiners.

Statistical analysis

The data were analyzed using Statistical Product and Service Solutions (SPSS, version 25.0; IBM SPSS Statistics for Windows, Armonk, NY). Normality was confirmed using the Shapiro-Wilk test (p > 0.05). Paired t-tests were used for within-group comparisons of pre- and post-staining measurements (stain retention and surface roughness). Between-group differences were assessed using one-way analysis of variance (ANOVA) with Tukey's post-hoc test for multiple comparisons, where significant (p < 0.05). All tests were two-tailed, with α = 0.05. Data are presented as mean and standard deviation (SD), with p < 0.05 considered statistically significant.

## Results

Comparative analysis of pre- and post-staining parameters revealed significant differences across all groups (Table 1). Both etching protocols demonstrated substantial stain absorption, with 37% phosphoric acid showing significantly higher retention than 10% hydrogen peroxide for both coffee (25.60 ± 2.10 vs. 18.20 ± 1.50 µg/mL; p < 0.001) and turmeric (16.80 ± 1.80 vs. 12.70 ± 1.20 µg/mL; p < 0.001) stains. The untreated control group exhibited intermediate staining values (8.30 ± 0.90 µg/mL for coffee, 5.10 ± 0.60 µg/mL for turmeric; p < 0.0001), suggesting that natural enamel porosity contributes to stain accumulation. These findings suggest that, while both methods enhance stain retention compared to untreated enamel, the more aggressive demineralization of phosphoric acid creates substantially more binding sites for chromogenic compounds.

**Table 1:** Comparison of stain retention rates between prfe- and post-staining in study groups across multiple stains with a paired t-test. *p < 0.05 denotes statistical significance using a paired t-test. Data are presented as mean and standard deviation. Absorption rate in µg/mL.

Groups	Type of stain	Absorption pre-staining (µg/mL)	Absorption post-staining (µg/mL)	t-value	p-value
10% hydrogen peroxide	Coffee	0.08 ± 0.01	18.20 ± 1.50	38.67	0.001*
	Turmeric	0.06 ± 0.01	12.70 ± 1.20	33.42	0.001*
37% phosphoric acid	Coffee	0.09 ± 0.01	25.60 ± 2.10	36.93	0.001*
	Turmeric	0.07 ± 0.01	16.80 ± 1.80	29.31	0.001*
Control	Coffee	0.05 ± 0.01	8.30 ± 0.90	29.11	0.0001*
	Turmeric	0.04 ± 0.01	5.10 ± 0.60	26.83	0.0001*

One-way ANOVA revealed highly significant differences in stain retention between groups (Table 2). For coffee staining, phosphoric acid etching showed the highest accumulation (25.51 ± 2.18 µg/mL), significantly greater than hydrogen peroxide (18.12 ± 1.45 µg/mL) and control (8.25 ± 0.82 µg/mL; p < 0.0001). A similar pattern was observed for turmeric staining. Post-hoc tests confirmed the following hierarchy: phosphoric acid > hydrogen peroxide > control (all p < 0.0001). The results demonstrate that etching dramatically enhances stain retention, with phosphoric acid's more aggressive demineralization creating approximately 40% more binding sites for chromogens than hydrogen peroxide.

**Table 2:** Comparison of absolute changes in stain retention rates between groups. *p < 0.05 denotes statistical significance using one-way analysis of variance. Data are presented as mean difference (absorption post-staining - absorption pre-staining).

Type of stain	Groups	Mean difference (µg/mL)	F-value	p-value	Post-hoc Tukey test
Coffee	10% hydrogen peroxide	18.12 ± 1.45	421.6	0.0001*	Phosphoric acid > Hydrogen peroxide > Control
	37% phosphoric acid	25.51 ± 2.18			
	Control	8.25 ± 0.82			
Turmeric	10% hydrogen peroxide	12.64 ± 1.14	228.9	0.0001*	Phosphoric acid > Hydrogen peroxide > Control
	37% phosphoric acid	16.73 ± 1.74			
	Control	5.06 ± 0.56			

The analysis revealed significant increases in surface roughness (Ra) staining across all groups (p < 0.05). Coffee caused greater roughness changes than turmeric in both groups. Notably, 37% phosphoric acid-etched surfaces showed the highest absolute roughness values post-staining (2.38 µm coffee, 2.05 µm turmeric). Even untreated controls demonstrated significant increases (+0.28 µm coffee, +0.08 µm turmeric). These findings indicate that both staining agents alter enamel topography, with coffee being more aggressive. Etching amplifies stain-induced roughness, particularly with phosphoric acid, and natural enamel remains vulnerable to surface changes (Table 3).

**Table 3:** Comparison of surface roughness between pre- and post-staining in study groups across multiple stains with a paired t-test. *p < 0.05 denotes statistical significance using a paired t-test. Data are presented as mean and standard deviation.

Groups	Type of stain	Surface roughness (Ra, µm) pre-staining	Surface roughness (Ra, µm) post-staining	t-value	p-value
10% hydrogen peroxide	Coffee	0.80 ± 0.20	1.18 ± 0.38	4.81	0.001*
	Turmeric	0.78 ± 0.20	0.92 ± 0.18	3.70	0.005*
37% phosphoric acid	Coffee	1.90 ± 0.30	2.38 ± 0.45	4.74	0.001*
	Turmeric	1.90 ± 0.30	2.05 ± 0.31	3.65	0.006*
Control	Coffee	0.30 ± 0.10	0.58 ± 0.18	5.89	0.001*
	Turmeric	0.30 ± 0.10	0.38 ± 0.14	2.81	0.022*

The one-way ANOVA revealed significant differences in coffee-induced surface roughness changes (p = 0.016), with both groups showing greater increases than the control (+0.28 µm) (p < 0.05). No significant differences were observed between etching agents (p > 0.05). For turmeric, roughness changes were minimal and statistically similar across groups (p = 0.13). These results suggest that coffee alters enamel roughness more aggressively than turmeric, etching exacerbates the abrasive effects of coffee, and the impact of turmeric is clinically negligible. Clinically, post-etching patients should avoid coffee to minimize enamel surface degradation, while dietary turmeric poses a minimal risk to surface integrity (Table 4).

**Table 4:** Comparison of absolute change in surface roughness between groups. p > 0.05 denotes no statistical significance using one-way analysis of variance. Data are presented as mean difference (surface roughness post-staining- surface roughness pre-staining).

Type of stain	Groups	Mean difference	F-value	p-value	Post-hoc Tukey test
Coffee	10% hydrogen peroxide	+0.38 ± 0.25	4.29	0.016	Phosphoric acid ≈ Hydrogen peroxide > Control (p < 0.05)
	37% phosphoric acid	+0.48 ± 0.32			
	Control	+0.28 ± 0.15			
Turmeric	10% hydrogen peroxide	+0.14 ± 0.12	2.15	0.13	No significant differences
	37% phosphoric acid	+0.15 ± 0.13			
	Control	+0.08 ± 0.09			

## Discussion

The findings of this study provided valuable insights into how common dental procedures and dietary chromogens influence enamel susceptibility to staining and surface alterations. The results indicated that both etching and bleaching significantly increased stain retention and surface roughness compared with the control group, with phosphoric acid etching demonstrating the most pronounced effects. These outcomes align with the hypothesis that enamel surface modifications, particularly those induced by etching, enhance the binding of chromogenic compounds and alter surface topography, with implications for dental aesthetics and enamel integrity.

The study found that enamel surfaces etched with 37% phosphoric acid exhibited significantly higher stain retention for both coffee and turmeric than hydrogen peroxide-treated surfaces and the control group. The increased stain retention in the etched groups can be attributed to the surface modifications induced by the etching agents. Phosphoric acid is commonly used in restorative dentistry to enhance bonding, remove the smear layer, and demineralize the enamel, creating a porous, honeycomb-like structure with an increased surface area [5]. This phenomenon of surface roughness is attributed to the depletion of minerals stemming from the dissolution of hydroxyapatite crystals present within the dental structure. The hydrophilic adhesive agent infiltrates the irregularities of the abraded tooth surface surrounding the etched hydroxyapatite crystals, thereby generating microtags [12]. This altered topography provides more binding sites for chromogenic compounds, such as curcumin in turmeric and caffeic acid derivatives in coffee, which are known to adhere to enamel through ionic and hydrophobic interactions.

Hydrogen peroxide, used in bleaching procedures, also modifies enamel by oxidizing organic components and causing subtle demineralization, although to a lesser extent than phosphoric acid [13]. Pereira et al. [14] reported no clinically detectable color change in surfaces etched with hydrogen peroxide. Etching was performed with 10% hydrogen peroxide for 15 and 60 seconds. The lower stain retention in the hydrogen peroxide group compared to that in the phosphoric acid group is likely due to its milder effect on enamel mineral content, resulting in fewer and less pronounced surface irregularities. Previous studies have shown that hydrogen peroxide bleaching increases enamel porosity but does not create extensive micro-roughness associated with phosphoric acid etching [8,9]. The control group, which received no etching treatment, exhibited the lowest stain retention, reflecting the natural protective barrier of intact enamel, which has a smoother surface and fewer chromogen binding sites.

Interestingly, coffee staining resulted in higher stain retention than turmeric staining across all groups. This may be due to the chemical composition of coffee, which contains polyphenolic compounds such as chlorogenic and caffeic acids that have a strong affinity for hydroxyapatite in enamel [15]. Turmeric, which is primarily composed of curcumin, is less polar and may have reduced binding efficiency compared with coffee polyphenolic compounds [3]. Manno et al. [16] reported that coffee led to thinning of the enamel layers, loss of continuity in the enamel-dentin junction, and wide spaces in dentin tubules.

However, a contrasting study by Telang et al. [17] suggested that turmeric caused more persistent staining than coffee owing to the intense pigmentation and stability of curcumin. This discrepancy could be attributed to the differences in the experimental conditions. In the present study, the standardized three-minute immersion protocol three times daily for seven days may have favored coffee’s staining potential due to its chemical properties, whereas prolonged exposure in Telang et al.'s [17] study might have enhanced the binding of curcumin.

The analysis revealed significant increases in surface roughness (Ra) post-staining across all groups, with coffee causing greater roughness changes than turmeric. The phosphoric acid-etched surfaces exhibited the highest post-staining roughness values, followed by the hydrogen peroxide-treated surfaces and the control group. These findings are consistent with the understanding that etching increases enamel surface irregularities, which are further exacerbated by staining agents [8,12]. Phosphoric acid creates a pronounced etching pattern by selectively dissolving enamel prisms, resulting in a rougher surface that is more susceptible to mechanical and chemical interactions with the staining agents [13]. The abrasive nature of coffee, attributed to its acidic pH and particulate matter, likely contributes to its greater impact on surface roughness compared to turmeric [15].

The control group also showed significant increases in roughness post-staining, suggesting that intact enamel is not immune to surface alterations from dietary chromogens. This is in accordance with the research conducted by Josey et al. [18], who indicated that the bleaching procedure led to a depletion of minerals from the enamel, which was observable 24 hours post-bleaching and persisted after 12 weeks of incubation in artificial saliva. Scanning electron microscopy revealed a significant alteration in the surface morphology of the bleached enamel. Furthermore, acid etching of the bleached enamel surface results in the loss of its prismatic structure, resulting in a markedly over-etched appearance of the enamel.

One-way ANOVA revealed significant differences in coffee-induced roughness changes between the etched and control groups, but no significant differences between the two agents. This suggests that, while etching amplifies the abrasive effects of coffee, the extent of the roughness increase is similar for phosphoric acid and hydrogen peroxide. A study by Torres-Gallegos et al. [19] reported the best etching results for 37% phosphoric acid for 15 seconds. The comparable roughness changes between these agents could be attributed to their shared ability to increase the surface porosity, albeit through different mechanisms.

Clinical implications

These findings have significant clinical implications for dental practice, particularly in aesthetic dentistry. Phosphoric acid etching, which is commonly used in bonding procedures for composite restorations, significantly increases enamel susceptibility to staining, especially in coffee. Clinicians should advise patients undergoing such procedures to avoid coffee consumption for at least 48-72 hours post-treatment, as this is when enamel is most vulnerable to chromogen binding. Hydrogen peroxide bleaching, while less aggressive, still increases stain retention and roughness, suggesting that patients should be counseled on post-bleaching dietary restrictions to maintain whitening results. This study also underscores the importance of patient education regarding dietary habits. The pronounced effect of coffee on both stain retention and surface roughness indicates that frequent consumption may compromise enamel integrity and aesthetics, particularly in patients with etched or bleached enamel. Turmeric, while less damaging, still contributes to staining, and patients with a high intake of turmeric-containing foods should be informed of its potential to affect dental aesthetics. To mitigate these effects, clinicians should consider protective measures, such as applying remineralizing agents (e.g., fluoride or casein phosphopeptide-amorphous calcium phosphate) post-etching or bleaching to reduce enamel porosity and enhance resistance to staining [20]. Additionally, polishing procedures after etching or bleaching may help restore a smoother enamel surface, thereby reducing the binding sites for chromogens.

Limitations

This study had several limitations. First, its in vitro design limits its ability to fully replicate the complex oral environment, including salivary flow, pellicle formation, and mechanical actions, such as brushing, which may modulate stain retention and surface roughness in vivo. Second, the seven-day staining protocol, while standardized, may not reflect long-term dietary exposure in the clinical setting. Third, the study focused on only two staining agents (coffee and turmeric), whereas other common chromogens, such as red wine or tea, may produce different effects. Finally, the use of flat enamel surfaces, while necessary for standardized measurements, does not account for the natural curvature and anatomical variations of teeth, which could influence staining patterns.

## Conclusions

This study demonstrated that phosphoric acid and hydrogen peroxide significantly enhanced enamel susceptibility to staining by coffee and turmeric, with phosphoric acid causing the most pronounced effects owing to its aggressive demineralization. Coffee induces greater surface roughness and stain retention than turmeric, highlighting its stronger impact on enamel topography and aesthetics. These findings emphasize the need for careful patient counseling and preventive strategies after etching or bleaching to maintain dental aesthetics. Future studies should explore in vivo conditions, additional chromogens, and the efficacy of remineralizing agents in mitigating these effects.
